# Meiotic Behaviors of Allotetraploid Citrus Drive the Interspecific Recombination Landscape, the Genetic Structures, and Traits Inheritance in Tetrazyg Progenies Aiming to Select New Rootstocks

**DOI:** 10.3390/plants12081630

**Published:** 2023-04-12

**Authors:** Lény Calvez, Alexis Dereeper, Aude Perdereau, Pierre Mournet, Maëva Miranda, Saturnin Bruyère, Barbara Hufnagel, Yann Froelicher, Arnaud Lemainque, Raphaël Morillon, Patrick Ollitrault

**Affiliations:** 1 UMR AGAP, CIRAD, F-97170 Petit-Bourg, France; leny.calvez@cirad.fr (L.C.); alexis.dereeper@ird.fr (A.D.); saturnin.bruyere@cirad.fr (S.B.); barbara.hufnagel@cirad.fr (B.H.); 2 UMR AGAP, Institut Agro, CIRAD, INRAE, University of Montpellier, F-34060 Montpellier, France; pierre.mournet@cirad.fr (P.M.); maeva.miranda@cirad.fr (M.M.); yann.froelicher@cirad.fr (Y.F.); raphael.morillon@cirad.fr (R.M.); 3 Genoscope, Institut de Biologie François-Jacob, Commissariat à l’Energie Atomique (CEA), Université Paris-Saclay, F-91000 Evry, France; aperdere@genoscope.cns.fr (A.P.);; 4 UMR AGAP, CIRAD, F-34398 Montpellier, France; 5 UMR AGAP, CIRAD, F-20230 San Giuliano, France

**Keywords:** polyploidy, rootstock breeding, meiosis, preferential pairing, recombination landscape, quantitative traits loci, citrus, *P. trifoliata*, *C. medica*, inter-specificity

## Abstract

Sexual breeding at the tetraploid level is a promising strategy for rootstock breeding in citrus. Due to the interspecific origin of most of the conventional diploid citrus rootstocks that produced the tetraploid germplasm, the optimization of this strategy requires better knowledge of the meiotic behavior of the tetraploid parents. This work used Genotyping By Sequencing (GBS) data from 103 tetraploid hybrids to study the meiotic behavior and generate a high-density recombination landscape for their tetraploid intergenic Swingle citrumelo and interspecific Volkamer lemon progenitors. A genetic association study was performed with root architecture traits. For citrumelo, high preferential chromosome pairing was revealed and led to an intermediate inheritance with a disomic tendency. Meiosis in Volkamer lemon was more complex than that of citrumelo, with mixed segregation patterns from disomy to tetrasomy. The preferential pairing resulted in low interspecific recombination levels and high interspecific heterozygosity transmission by the diploid gametes. This meiotic behavior affected the efficiency of Quantitative Trait Loci (QTL) detection. Nevertheless, it enabled a high transmission of disease and pest resistance candidate genes from *P. trifoliata* that are heterozygous in the citrumelo progenitor. The tetrazyg strategy, using doubled diploids of interspecific origin as parents, appears to be efficient in transferring the dominant traits selected at the parental level to the tetraploid progenies.

## 1. Introduction

Citrus cultivation is one of the major fruit productions in the world. However, this production is confronted with numerous biotic and abiotic constraints, including drought, extreme temperature, salinity, and pest and disease. These stresses devastate orchards by severely influencing tree growth and development, fruit production, and quality. Rootstocks are widely used for citrus cultivation because they improve trees’ performance and aim at tolerance or even resistance to various stresses. Citrus breeding programs are essential to create new rootstock varieties better adapted to current global issues, such as environmental pressures exacerbated by global warming [1] and the emergence of certain diseases such as Huanglongbing (HLB). Due to a sizeable sexual compatibility within the “true citrus” gene pool as defined by [2], many natural or man-driven interspecific and even intergeneric hybridizations resulted in interesting rootstocks [3]. Volkamer lemon (*Citrus limonia* Osb.) is one of the parental rootstocks used in the present work. It is an example of a natural interspecific F1 hybrid between a mandarin (*C. reticulata* Blanco) and a citron (*C. medica* L.) [4,5]. Used as rootstock, Volkamer lemon confers strong growth and good fruit yield. It is adapted to dry, calcareous, and saline soil and presents good tolerances against *Citrus tristeza virus* (CTV), exocortis viroid, and *Phytophthora* spp. [6].

Conventional citrus breeding by sexual hybridizations has been practiced since the end of the 19th century and is still commonly used today. Among the citrus relatively sexually compatible with the *Citrus* genus [7], *Poncirus trifoliata* (L.) Raf. has good agronomic qualities that make it a crucial genetic resource in citrus rootstock improvement programs. Indeed, *P. trifoliata* is resistant to nematodes [8] and CTV, which is the most critical citrus viral disease [9]. *P. trifoliata* is highly tolerant to *Phytophtora* spp. [10] and cold temperatures [11]. In addition, it has been described as tolerant to HLB [12,13,14,15,16,17], but this observation is still controversial [13]. However, *P. trifoliata* is susceptible to alkaline soils and salinity, which limits its use in many citrus-growing regions [7,18]. As a seedling, *P. trifoliata* genotypes are quite tolerant to water deficit [19]. Intergeneric hybridizations between *Poncirus* and *Citrus* genera allow the combination of several traits. Intergeneric hybridization (*Poncirus* × *Citrus*) seems to be an efficient way to develop new rootstock genotypes responding to the new challenges of the citrus industry. New intergeneric citrus hybrids such as Swingle citrumelo (*C. paradisi* Macf. × *P. trifoliata* (L.) Raf.), citrandarin (*C. reticulata* × *P. trifoliata*) Carrizo, Troyer, and C35 citrange (*C. sinensis* (L.) Osb. × *P. trifoliata*) have good agronomic properties and are widely used around the world [6]. The Swingle citrumelo rootstock, one of the genotypes used in this work, is resistant to nematodes, *phytophthora,* and CTV and also confers a good cold tolerance [6].

Most of the citrus germplasm and worldwide-used citrus rootstocks are diploid. However, during the last decade, several studies have shown that tetraploid citrus rootstock, compared to their diploid counterpart, showed increased tolerance to different pedoclimatic constraints such as salt stress [20,21,22,23], low temperature [24], nutrient deficiency [25,26], and water deficit [27,28,29]. Sivager et al. (2022) showed that tetraploid Swingle citrumelo rootstocks improve HLB tolerance in diploid and triploid grafted scions. Interestingly, the better tolerance in polyploid root and the scion is related to maintaining the phloem sap flux by limiting the callose deposition [30] and a capacity to cope more effectively with the oxidative stress induced by the disease at the leaf level [30] and the root level [31]. As a consequence of this finding, ploidy manipulations have become an integral aspect of citrus breeding programs [32].

Polyploids can result from sexual or somatic polyploidization events. Citrus sexual polyploidization mainly results in the formation of triploids through the union of unreduced gametes with normal haploid gametes [33,34,35]. Due to the seedlessness of most triploid hybrids, sexual polyploidization is actively exploited for seedless variety breeding [36] but not for rootstock breeding. Somatic polyploidization is frequently observed in apomictic citrus species, with a chromosomal doubling of nucellar tissues resulting in spontaneous doubled diploid genotypes [37,38]. Doubled diploids can also be artificially induced by chemical treatment with colchicine or oryzalin [39,40,41]. Several teams have widely used somatic hybridization to create tetraploid rootstocks combining characters of interest from different genetic resources. This method allows researchers to add genomes of two diploid genotypes without sexual recombination [42,43,44,45,46,47,48,49] and, therefore, to combine favorable dominant traits of the two parents. Recent genomic studies revealed chromosome instability in somatic hybrids, which can lead to substantial loss of genomic regions of parental lines [42]. 

Another way to combine the diversity of two parents for tetraploid rootstock breeding is sexual hybridization between two tetraploid hybrids. This “tetrazyg” breeding strategy was successfully used to select some new tetraploid hybrid rootstocks [49,50,51,52].

It is essential to develop knowledge of the mode of inheritance in tetraploid hybrids to optimize the efficiency of the “tetrazyg” strategy. Tetraploids can be distinguished as autotetraploids or allotetraploids based on the composition of their genomes [53,54]. Autotetraploids are derived from genomic material from a single species, while allotetraploids result from combining genomes from different species. The meiotic behavior of tetraploids, particularly the transmission of parental heterozygosity, depends significantly on the phylogenomic structure of the polyploid. Thus, two extreme models are considered: disomy in allotetraploids and tetrasomy in autotetraploids. In allotetraploids, there are two diploid sets of homologous chromosomes. During meiosis, homologous chromosomes are preferentially paired, and only bivalents are formed [53]. This results in a disomic inheritance that, in the extreme case, allows a total restitution of the interspecific parental heterozygosity [55]. In autotetraploids, there are four homologous versions of each chromosome. During meiosis, there is a random pairing with any of its homologs, forming bivalents or quadrivalents. This configuration leads to tetrasomic inheritance, where all allelic combinations are produced [55]. Heterozygosity restitution ranges from 66% for the extreme tetrasomic pattern, in the absence of double reduction, to 55% in the presence of double reduction [56]. In cases where parents are divergent but have retained enough homology to prevent exclusive preferential pairing, chromosome pairing is neither exclusively preferential nor entirely random, and intermediate patterns of inheritance between di- and tetrasomic are expected [5,18,53,55,57]. Such intermediate inheritance enables the diploid gamete to transmit an important part of the interspecific parental heterozygosity and provides opportunities for interspecific recombination [3]. Intermediate inheritance with disomic tendency has been described in several tetraploid hybrids resulting from somatic hybridization between diploid *C. reticulata* and *P. trifoliata* [18] or chromosome doubling of diploid *Citrus × Poncirus* intergeneric hybrids [3,18]. Intermediate inheritance was also observed for tetraploid hybrids resulting from chromosome doubling of an interspecific *C. micrantha* Wester × *C. medica* L. hybrid [57]. 

The present study focuses on a tetraploid hybrid population obtained by sexual crossing between a tetraploid Swingle citrumelo and a tetraploid Volkamer lemon. Both tetraploid parents result from chromosome doubling of the corresponding diploid germplasm of intergeneric (*C. paradisi* × *P. trifoliata*) and interspecific origins (*C. reticulata* × *C. medica*). Therefore, both should be considered allotetraploid. According to the agronomic behavior of the diploid rootstock lines described above and their complementarity, it is expected that the hybridization between these two tetraploid parents could help create new rootstocks corresponding to the demand of citrus growers.

The first study of the meiotic behavior of the tetraploid Swingle citrumelo was performed in this population by analyzing the segregation of 159 SNP markers that fully distinguished the genus *Poncirus* from the genus *Citrus* [3]. It revealed an intermediate inheritance with disomic tendency, allowing intergeneric recombination and opening the opportunity for phenotype–genetic association studies. 

In the present work, we used a GBS approach anchoring on the *P. trifoliata* reference genome (https://phytozome.jgi.doe.gov/; accessed on 3 Marsh 2022) for simultaneous SNP identification and high-throughput genotyping of the tetraploid population. Diagnostic SNPs for the four ancestral species constitutive of the parents (*C. reticulata*, *C. maxima*, *C. medica,* and *P. trifoliata*) were identified, and the TraceAncestor software [5] was applied to analyze the phylogenomic structure of each hybrid. This analysis permitted the identifications and locations of the interspecific (*C. reticulata/C. medica*) and intergeneric (*Citrus/Poncirus*) recombination points for the Swingle citrumelo and Volkamer lemon, respectively. Linkage maps and recombination landscapes were established, as well as the preferential pairing and the parental heterozygosity restitution along the genome for the two parents.

Size, density, length, proliferation, expansion, and growth rate of roots are essential characteristics for a good adaptation to drought and an important component for less impact of HLB disease during the dry season. We, therefore, performed phenotyping to evaluate the root development of each hybrid to characterize and select the genotypes with the best root system conformation. These phenotypic data were coupled with GBS genotyping data to search for QTL responsible for root elongation. Finally, we studied the inheritance in each hybrid of some genes and QTL of interest previously located in the *P. trifoliata* genome by [58].

## 2. Results

### 2.1. DSNP Mining 

DSNPs were identified from GBS data of eight accessions representative of the basic *Citrus* and *Poncirus* diversity. After filtering with less than 20% of missing data, the VCFHunter pipeline delivered 144,871 dialelic SNPs. With a GST value of 1, we identified a total of 72,452 DSNPs along the 9 chromosomes, including 9444 DSNPs for *C. maxima*, representing 13.03% of the total number of DSNPs. A total of 13,941 DSNPs were found for *C. reticulata* (19.24%), 21,230 for *C. medica* (29.30%), and 27,837 for *P. trifoliata* (38.42%). All the detailed information on DSNPs is provided in Table 1.

### 2.2. Genetic Linkage Maps of Tetraploid Swingle Citrumelo and Volkamer Lemon

GBS data from tetraploid rootstock hybrids were filtered with less than 20% missing data, and VCFHunter software identified 212,072 polymorphisms. For genetic mapping, data were filtered with less than 15% missing data for genomic windows (of 20 DSNPs) and less than 20% for individuals. The method we used to estimate the doses is based on assuming the physical ordering is correct. To generate the genetic map, we used a matrix of 1318 genomics windows and 95 hybrids for the tetraploid Swingle citrumelo and 1016 genomics windows and 96 individuals for Volkamer lemon. Marker-pairwise recombination frequencies were calculated, and nine linkage groups were identified during the linkage grouping constitution. Without preconceptions, we found that all the windows of the same chromosome were localized in the same linkage group. Thus, it confirms the total synteny between linkage groups and physical assembly (Supplementary Figures S1 and S2). In the Swingle citrumelo genetic map, the number of mapped windows varied from 116 (LG6 and LG9) to 236 (LG3). The genetic map’s total size was 435.64 cM, ranging from 28.20 cM (LG8) to 98.46 cM (LG3) (Table 2). In the Volkamer lemon genetic map, the number of mapped windows varied from 94 to 187 for LG6 and LG3, respectively. The total length of the map was 425 cM, with the smallest linkage group of 20.10 cM (LG8) and the largest one of 105.41 cM (LG3) (Table 2).

### 2.3. Recombination Rate

**Figure 1:**
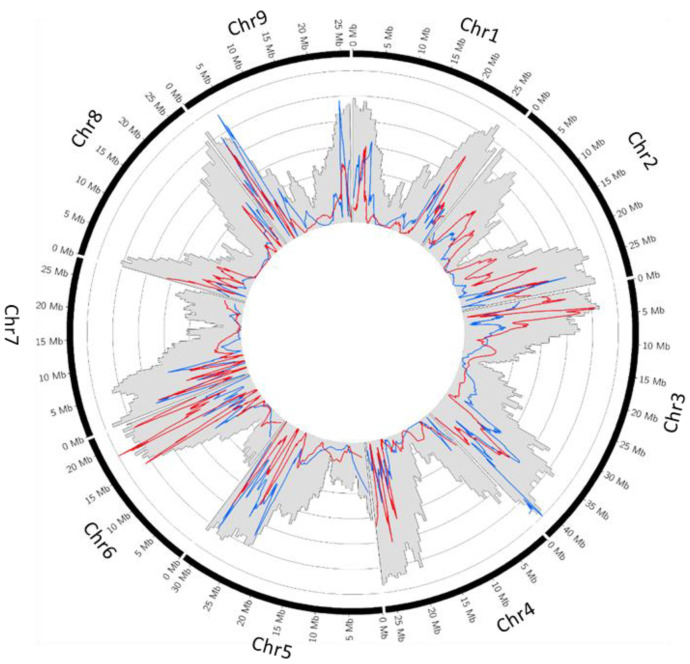
Circos plot of the recombination rate along the nine chromosomes. The red lines represent the recombination rate of the Swingle citrumelo and the blue lines Volkamer lemon (Min = 0; Max = 11.72 cM/Mb). The grey histogram represents the gene density in the *P. trifoliata* reference genome (Min = 0; Max = 0.57).

The recombination rates for tetraploid Swingle citrumelo and tetraploid Volkamer lemon were estimated and visualized in Circos (Figure 1). 

For all chromosomes, we observed a close link between the gene density in the *P. trifoliata* reference genome and the recombination rate of Swingle citrumelo and Volkamer lemon gametes with a decreasing recombination rate around the centromeric regions. Comparison between recombination rates of Swingle citrumelo and Volkamer lemon gametes showed, for chromosome 1, predominantly greater recombination values for Volkamer lemon gametes, except at the chromosome end, where the recombination rate was greater in Swingle citrumelo. Chromosomes 2, 4, 6, and 8 exhibited higher recombination values in Swingle citrumelo. However, the recombination rates over chromosomes 5 and 9 were lower in Swingle citrumelo than Volkamer lemon. For chromosome 3, higher recombination values were observed for Swingle citrumelo between the beginning of the chromosome and the centromere, while, for the other chromatid of the chromosome, the recombination rate was higher in Volkamer gametes. For chromosome 7, a high recombination rate was recorded at the beginning and a low value at the end of the chromosomes, with a similar profile between both genetic maps. For both parents, we observed a direct association between low combination rates and centromeric and pericentromeric genomic regions that also exhibit low gene sequence density on the *P. trifoliata* genome [58].

**Figure 2:**
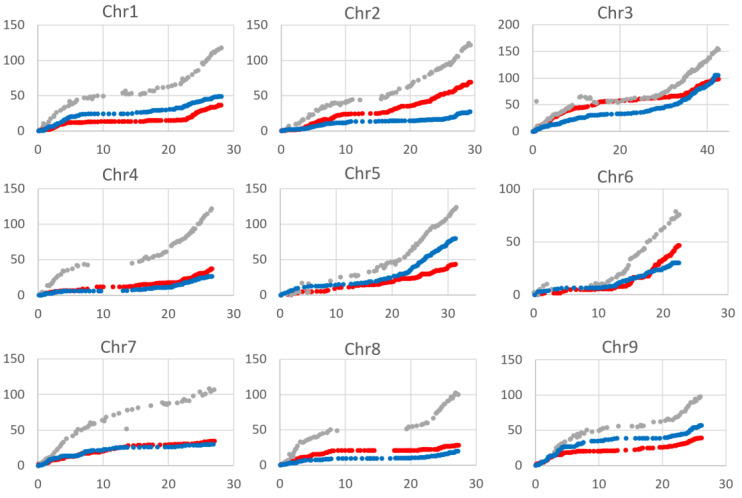
Marey map plot of the nine linkage groups of the Swingle citrumelo genetic map (red dot), Volkamer lemon genetic map (blue dot), and trifoliate orange reference genetic map generated in Huang et al. (2018; [59]) (grey dot). The x-axis represents the physical positions (in Mb) on the *P. trifoliata* reference genome (Peng et al. 2020; [58]), and the y-axis represents the position on each genetic map (in cM).

The variations of the estimated recombination rates agree with the Marey maps presented in Figure 2.

The genetic positions anchored on the physical position of the *P. trifoliata* reference genome [58] were represented for the Swingle citrumelo, Volkamer lemon, and trifoliate orange reference [59] genetic maps. A high recombination rate results in larger genetic distances by physical unit. We observe that the *P. trifoliata* reference genetic map is much larger for all chromosomes. Plateaus corresponding to very low recombination rates were observed in the three maps “for analogous regions of the *P. trifoliata* genome.”

### 2.4. Parental Heterozygosity Restitution and Meiotic Inheritance Analysis

The Parental Heterozygosity Restitution (PHR) and Preferential chromosome Pairing (PP) were analyzed for both Swingle citrumelo and Volkamer lemon parents along the genome. We used the same matrix we used for genetic mapping to estimate PHR and PP. PHR was determined for each genomic window and chromosome, and PP was calculated from three markers located around the centromere, according to [56,60]. 

**Figure 3:**
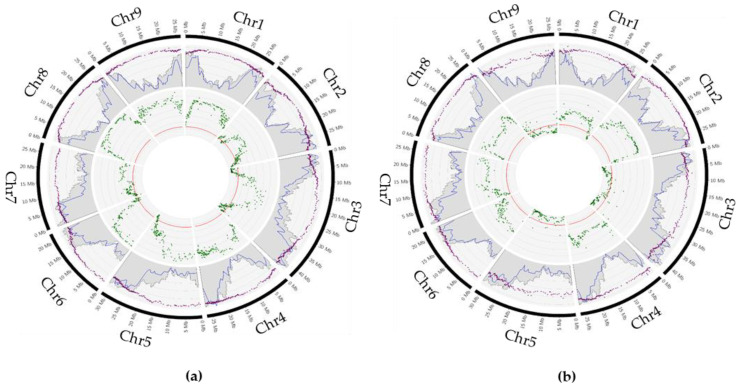
Circos plot of meiotic parameter in (**a**) Swingle citrumelo and (**b**) Volkamer lemon gametes along the nine chromosomes. In the first section: violet dots in the scatter plot represent the Parental Heterozygosity Restitution; grey histograms represent the gene sequence density in the *P. trifoliata* reference genome (Peng et al. 2020; [58]), and the blue lines represent the marker density. In the inter section: green dots represent the q-value for the deviation to the tetrasomic gametic segregation model and the red lines represent the limit of significant value.

For the diploid Swingle citrumelo gamete, the average PHR over the 1318 genomic windows and the 95 gametes was 85.9%. PHR varied between chromosomes from 0.790 ± 0.007 for chromosome 3 to 0.933 ± 0.006 for chromosome 8. Significant differences in PHR between chromosomes were revealed by a Kruskal–Wallis test (*p*-value < 2.2 × 10^−16^) followed by a Wilcoxon test (*p*-value < 0.05) (Figure 3a).

The average PP was 76.3%. Significant PP variations between chromosomes were confirmed by a one-way ANOVA (*p*-value < 4.12 × 10^−15^) followed by a Newman–Keuls test (*p*-value < 0.05). Chromosomes 6 and 8 had the highest PP values, with 0.925 ± 0.086 and 0.923 ± 0.050, respectively. PP rates are significantly similar for chromosomes 4 and 5, with 0.850 ± 0.043 and 0.848 ± 0.047, and chromosomes 9 and 1, with 0.760 ± 0.045 and 0.742 ± 0.007, respectively. Chromosome 3 had a PP value of 0.690 ± 0.054, followed by chromosome 2 with 0.625 ± 0.033, and chromosome 7 had the lowest PP rate of 0.502 ± 0.029 (Table 3). 

The results for the deviation to the tetrasomic gametic segregation model at the individual window level based on q-value [61] are in accordance with previous analysis (Figure 3a). Among the 1318 windows, 1109 (84.14%) showed significant segregation distortion. Low frequency of significant distortions to the gametic tetrasomic segregation were observed in chromosome 3 (45.76%). The other chromosomes presented a high value of significant gametic segregation distortion, ranging between 72.09% (chromosome 2) and 100% (chromosomes 1, 4, 8, and 9). The lowest average values were observed for chromosomes 3, 2, and 7, with 1.634 ± 0.171, 1.874 ± 0.129, and 2.084 ± 0.102. The highest average value was found in chromosomes 4, 8, and 9 (−log(q-value) > 5) (Table 4a). On the whole, Swingle citrumelo showed an intermediate inheritance with a disomic tendency for all chromosomes and particularly high for chromosomes 6 and 8.

For the Volkamer lemon diploid gamete, the average PHR over the 1016 genomic windows and the 96 gametes was 81.7%. PHR varied between markers from 0.692 ± 0.008 to 0.911 ± 0.008, and significant differences in PHR between chromosomes were revealed by a Kruskal–Wallis test (*p*-value < 2.2 × 10^−16^) followed by a Wilcoxon test (*p*-value < 0.05). The average PP for Volkamer lemon was 50.2%. Significant PP variations between chromosomes were confirmed by a one-way ANOVA (*p*-value < 2.2 × 10^−16^) followed by a Newman–Keuls test (*p*-value < 0.05). Chromosome 2 had the highest PP value of 0.810. Chromosomes 6, 8, and 7 had PP values significantly similar, from 0.675 ± 0.012 to 0.663 ± 0.036. Chromosomes 1 and 4 showed similar PP rates of 0.583 ± 0.019 and 0.548 ± 0.061. Chromosome 3 had a PP value of 0.317 ± 0.061, followed by chromosome 9 with 0.250 ± 0.054, and chromosome 5 with the lowest PP rate of 0 (Table 3). From the 1016 windows, 664 (65.35%) showed significant deviation of gametic tetrasomic segregation. A low percentage of significant tetrasomic inheritance deviation was observed for chromosomes 5 (6.67%), 3 (9.63%), and 9 (24.74%). Chromosome 1 displayed 88.68% of windows with such significant deviation. All the windows presented significant deviation for all the other chromosomes (chromosomes 2, 4, 6, 7, and 8). The lowest average q-values were observed for chromosomes 5, 3, and 9, with 0.407 ± 0.171, 0.549 ±0.129, and 0.959 ± 0.157. The highest average values were found in chromosomes 2 and 8, with a q-value over 4 (Table 4b). Volkamer lemon presented intermediate segregation throughout, with critical inter-chromosomal differences (Figure 3b). Chromosomes 1, 2, 4, 6, 7, and 8 showed a disomic tendency, while chromosomes 3 and 9 displayed a tetrasomic tendency. Chromosome 5 exhibited a fully tetrasomic inheritance behavior.

A two-way ANOVA (*p*-value < 2.2 × 10^−16^) followed by a Newman–Keuls test (*p*-value < 0.05) were conducted to compare PP values between Swingle citrumelo and Volkamer lemon gamete populations. Chromosomes 1, 3, 4, 5, 6, 8, and 9 had significantly higher PP in Swingle citrumelo, while chromosomes 2 and 7 had higher PP in Volkamer lemon (Supplementary Figure S3). 

### 2.5. Impact of Preferential Pairing on Tetraploid Hybrid Diversity Structure

A factorial analysis on the dissimilarity matrix was performed to investigate the genetic distance of the hybrid population from parentage and other genotypes representative of *Citrus* and *Poncirus* diversity. Our goal was to analyze the meiotic behavior and its implication on hybrid population diversity. As we have previously identified interesting preferential pairing differences between chromosomes 2 and 5, we focused our study on these two.

**Figure 4:**
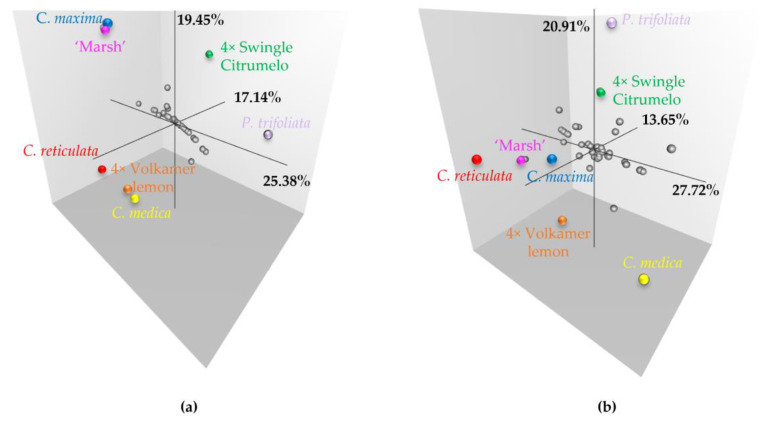
Factorial analysis based on a dissimilarity matrix established from ancestral doses along the genome for (**a**) chromosome 2 and (**b**) chromosome 5. Color dots represent parentage and grey dots represent the Volkamer lemon × Swingle citrumelo tetraploid hybrids.

For chromosome 2, factorial analysis (Figure 4a) was represented on three axes (first axis: 25.38%, second axis: 19.45%, and third axis: 17.14%).

The first axis opposes the *Citrus* species and the *Poncirus* species. Tetraploid Swingle citrumelo and Volkamer lemon were differentiated by the second axis, with the intergeneric hybrids in intermediate positions. Hybrids were extended with a large diversity between *P. trifoliata* and ‘Marsh’ pummelo, which corresponds to the recombination of Swingle citrumelo. For this chromosome, Volkamer lemon displayed a high value of preferential pairing, which limits recombination events.

For chromosome 5, factorial analysis (Figure 4b) showed a first axis (27.72%) as opposed to *C. reticulata* and *C. maxima* against *C. medica*. The second axis primarily differentiated *Poncirus* and *Citrus* genotypes (20.91%). Hybrid orientation was done on the first axis with important diversity between *C. reticulata* and *C. medica*, which correspond to the recombination of Volkamer lemon. The null preferential paring estimated in Volkamer lemon leads to increased *C. medica/C. reticulata* recombination rates and Volkamer gamete diversity.

### 2.6. Inheritance in the Tetraploid Hybrids of Candidate Genes for Pest and Disease Resistance 

The inheritance in the hybrid population was analyzed for the candidate genes associated with HLB tolerance, CTV resistance, and nematode resistance identified by Peng et al. (2020) [58]. The localization of these genes on the *Poncirus* genome was determined, and we analyzed the phylogenomic structure of hybrids at these positions. As the tolerance traits come from the *Poncirus* ancestor, hybrids carrying the *Poncirus* haplotype have potentially inherited the favorable trait carried by the candidate gene. 

According to Huang et al. (2018) [59], HLB tolerance is associated with 11 genes localized in scaffolds 6 and 9. In scaffold 6, 4.21% of the hybrids did not inherit the *Poncirus* genome on the positions of the candidate gene, 83.16% had one dose of *Poncirus*, and 9.47% of the hybrids inherited two doses of *Poncirus. Poncirus* dose remained undetermined for 3.16% of the hybrids (Table 5).

In scaffold 9, 10 candidate genes are localized in two regions where 5.26% and 4.21% of the hybrids did not inherit the *Poncirus* genome on the desired positions, 90.53% and 88.42% had one dose of *Poncirus,* and 1.05 and 2.11% of the hybrids inherited two doses of *Poncirus*. *Poncirus* doses remained undetermined for 3.16% and 5.26% of the hybrids. 

Four CTV resistance candidate genes are localized in scaffold 7 (11780276–11912236). For the positions of interest, 10.53% of the hybrids did not inherit *Poncirus*, 77.89% inherited one dose, and 9.47% two doses of *Poncirus*. In addition, 2.11% remained undetermined.

Fifteen candidate genes for nematode resistance are localized in a 1 Mb region of scaffold 7 of the *Poncirus* genome, and 11.58% of the hybrids did not inherit the *Poncirus* genome. According to the genes, between 76.84% and 78.95% of the hybrids inherited one dose and 7.37% to 9.47% inherited two doses of *Poncirus.* Indetermination concerned between 0 and 2.11% of the hybrids. 

A total of 77.89% of hybrids have inherited at least one dose of *Poncirus* for all genomic regions considered.

### 2.7. Root Phenotyping and Genomic Association

**Figure 5:**
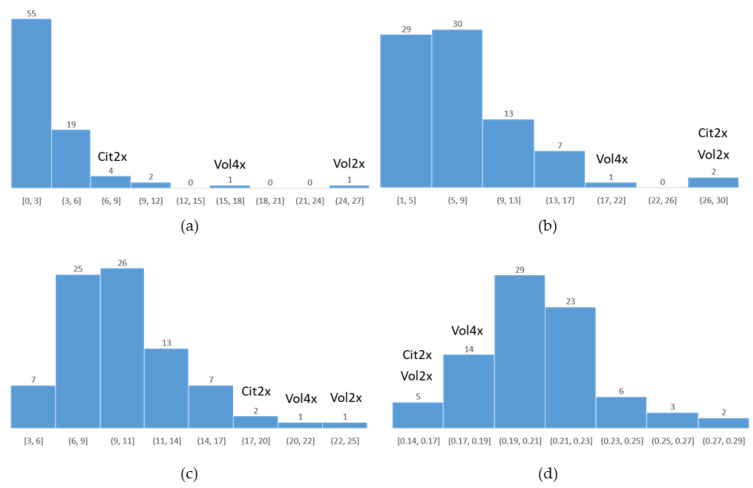
Distribution of the four root phenotypic traits in the 4× Volkamer lemon × 4× Swingle citrumelo progenies. (**a**) Number of secondary roots; (**b**) root surface; (**c**) root length; (**d**) root diameter.

The root length, root area, number of secondary roots, and root diameter were evaluated in the population. Unfortunately, we were not able to propagate cuttings of Swingle citrumelo 4×. The measurements were highly heterogeneous between hybrids, and we observed a large diversity in root system morphology (Supplementary Figure S4). Statistical analyses using linear-mixed effects models showed a significant effect of genotype (*p*-value < 0.001) on the different measured traits. The hybrid values were distributed mainly outside the Swingle citrumelo 2× and Volkamer lemon 2×/4× range (Figure 5).

The tetraploid hybrids displayed mostly lower values than the diploid and tetraploid parents regarding the number of secondary roots, root surface, and root length, while showing greater root diameters. A PCA analysis confirmed the relationships between the different measured variables (Supplementary Figure S5). The first axis, explaining 46.67% of the variables, is characterized by root area, number of secondary roots, and root length. The arrows related to these variables are clustered around the first axis, so these traits are highly correlated. Root diameter drives the second principal axis, explaining 21.24% of the root variables. However, on axis 1, this variable also seems opposed to root length.

**Figure 6:**
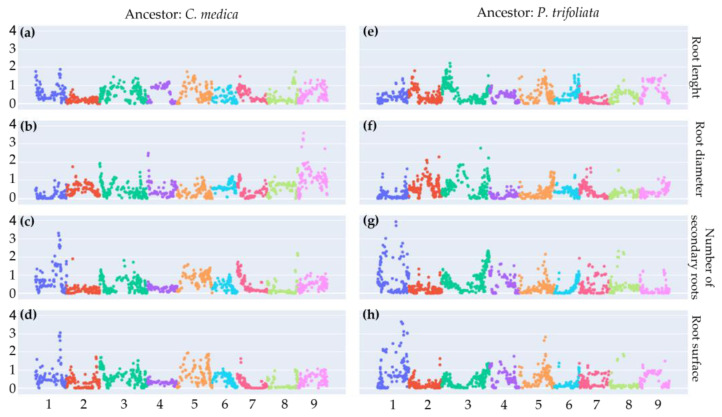
Evaluation of the association between ancestors doses in diploid gametes and root traits. Manhattan plots from GLM procedure: X axis: position on the nine *P. trifoliata* chromosome; Y axis: −log_10_(*p*-value); (**a**,**e**): root length, (**b**,**f**): root diameter, (**c**,**g**): number of secondary roots, (**d**,**g**): root surface. (**a**–**d**): *C. medica* doses in Volkamer lemon gametes. (**e**–**h**): *P. trifoliata* allelic doses in Swingle citrumelo gametes.

With these phenotyping data, we tried to identify some genetic determinants of the root system architecture in the hybrids through genetic association analyses. We used ancestral doses of *C. medica* and *P. trifoliata* in the diploid gamete populations of Volkamer lemon and Swingle citrumelo that generated our tetraploid hybrids. A significant genome association signal (*p*-value > 0.01) was observed for the two gamete populations at the end of chromosome 1 (Figure 6) for the number of secondary roots and root surfaces.

Another significant signal was noticed for the Volkamer lemon gamete at the beginning of chromosome 9 for root diameter. In addition, a weaker significant signal was detected for the Swingle citrumelo gamete at the end of chromosome 5 for the root surface trait.

Considering the marker with the lower *p*-value, the boxplot analyses of the hybrids (Supplementary Figure S6) reveal a positive effect of the *P. trifoliata* haplotype and a negative effect of *C. medica* for the three traits in all identified regions. However, the distribution of the different classes of ancestral doses (zero, one, or two) in the diploid gametes is highly unbalanced, with a strong excess of one dose (heterozygous gametes) resulting from the intermediate segregation of the two allotetraploid parents.

## 3. Discussion

### 3.1. GBS Coupled with TraceAncestor Was Efficient for Estimating Haplotype Ancestral Doses All along the Genome of the Tetraploid Hybrids

The genetics of polyploid species are much more complex than diploid ones. In particular, the accurate study of polyploid populations requires the estimation of the allelic copy number for heterozygous genotypes. Allele dosage can affect gene expression and, therefore, the phenotype. Their determination is, therefore, essential for genotype/phenotype association studies [62]. Various methods for estimating allelic dosage in citrus polyploid plants have been developed using co-dominant markers. For Simple Sequence Repeat (SSR) markers, the MAC-PR method [63], proposed to deal with differential amplification intensities between alleles, has been successfully used in citrus [34,64]. The KASPar genotyping technique, based on competitive allele-to-allele PCR, is also suitable for studying SNP marker allelic patterns within polyploid populations [65]. The KASPar genotyping technique has been successfully applied for the analysis of the origin of 2n gametes and their genetic structures [33,35,60,66] as well as for the analysis of the meiotic behavior of tetraploid progenitors [3,18,56,57,67,68]. 

However, the two previous methods are adapted for studies requiring a limited number of markers, but not for high throughput analysis. Taking advantage of Next Generation Sequencing (NGS), the authors of [5] developed a likelihood method based on the multilocus number of ancestral reads of successive phased markers to estimate the contribution of ancestral haplotypes in diploid and polyploid admixed plants. Our tetraploid population is the result of crossing tetraploid Swingle citrumelo (*C. paradisi* × *P. trifoliata*) and Volkamer lemon (*C. medica* × *C. reticulata*) rootstocks [3]. According to recent phylogenomic studies, the grapefruit parent (*C. paradisi* Macf.) of Swingle citrumelo results from hybridization between a pummelo (*C. maxima*) and a sweet orange (*C. sinensis*), sweet orange being itself an admixture between *C. reticulata* and *C. maxima* [5,69,70]. Therefore, our Volkamer lemon × citrumelo hybrids have a complex genetic structure with inheritance from four ancestral parents, including *C. medica*, *C. reticulata*, *C. maxima*, and *P. trifoliata*. From our GBS study, we identified 72,452 DSNPs of these 4 ancestral species along the 9 chromosomes: 9444 DSNPs for the *C. maxima* ancestor, 13,941 DSNPs for *C. reticulata*, 21,230 for *C. medica*, and 27,837 for *P. trifoliata*. Using the TraceAncestor tool [5], the estimation of the allelic doses of the *C. medica* and *P. trifoliata* ancestors in each tetraploid hybrid allowed us to deduce the phylogenomic structure of the corresponding Volkamer lemon ovule and Swingle citrumelo pollen that generate each hybrid and, therefore, to identify and locate the interspecific (*C. reticulata*/*C. medica*) and intergeneric (*Citrus*/*Poncirus*) recombination events, respectively, for the Volkamer lemon and Swingle citrumelo parent.

### 3.2. The Tetraploid Volkamer Lemon and Swingle Citrumelo Parents Display Intermediate Inheritance with a Disomic Tendency and Variability between Parents and Chromosomes for Preferential Chromosome Pairing

According to [56], we analyzed preferential pairing in the Swingle citrumelo and Volkamer lemon using three centromeric and pericentromeric genetic windows with the assumption of no double reduction. In addition, Parental Heterozygosity Restitution (PHR) of the diploid gamete was computed for each marker. For most of the genome, intermediate segregation with a clear disomic tendency was identified in Swingle citrumelo (PHR: 0.859 ± 0.004; PP: 0.763 ± 0.054). Volkamer lemon displayed an intermediate inheritance with slight disomic behavior (PHR: 0.817 ± 0.005; PP: 0.502 ± 0.099). Such intermediate inheritance with a disomic tendency has already been described in the ‘Giant key’ doubled-diploid Mexican lime [57,67] and in Flhorag1 allotetraploid rootstock [18]. Inter-chromosomal comparisons revealed significant differences in PP and PHR, resulting in segregation pattern differences between chromosomes. 

For tetraploid Swingle citrumelo, LG7 displayed a clear intermediate inheritance (PP = 0.502). Furthermore, other LGs (LG1, LG2, LG3, LG4, LG5, LG6, LG8, and LG9) displayed an intermediate inheritance with a disomic inheritance tendency (PP comprised between 0.625 and 0.925). Disomy was particularly marked for LG6 and LG8, with more than 92% preferential chromosome pairing.

For tetraploid Volkamer lemon, a complete tetrasomic inheritance was identified for LG5 with an absence of PP. LG3 and LG9 showed an intermediate inheritance with a tendency towards a tetrasomic inheritance (PP [LG9] = 25%; PP [LG3] = 32%). LG1 and LG4 presented a clear intermediate inheritance with PPs of 58% and 55%, respectively. LG2, LG6, LG7, and LG8 exhibited an intermediate inheritance with a tendency towards a disomic inheritance with PPs between 66% and 81%.

Such inter-chromosomal differences in segregation pattern have already been observed in a doubled-diploid clementine displaying tetrasomic segregation, intermediate segregation with a tetrasomic tendency, and intermediate segregation with a disomic tendency [56] and in a citrus allotetraploid somatic hybrid (‘Nova’ tangelo + ‘HB’ pummelo) that displayed a mixed disomic, tetrasomic, and intermediate inheritance [71]. In addition, this kind of segregation was observed in other species, such as in colchicine-induced allotetraploid *Musa* [72].

### 3.3. Apparent Interspecific and Intergeneric Recombination Rates Are Limited by Preferential Pairing and Are Very Low in Centromeric-Pericentromeric Areas

Despite predominant disomic inheritance, we observed interspecific recombination in the nine citrus chromosomes for the two allotetraploid parents, which allowed us to establish the two parental genetic maps. The construction of high-density genetic maps is valuable for breeders because it will potentially facilitate genomic studies and identify genomic regions with interesting agronomic traits in citrus. Our genetic maps have been anchored on the *P. trifoliata* reference genome [58]. During the elaboration of the genetic maps, we observed that all the windows of the same chromosome were in the same linkage group. This observation confirms the total synteny of the genetic linkage maps with the *P. trifoliata* reference genome. Due to the quality of the *P. trifoliata* assembly and the collinearity that has already been demonstrated between the genus *Citrus* and *Poncirus* [3,59,73,74,75], we assumed that the physical ordering of *P. trifoliata* was the correct ordering. For Swingle citrumelo, the genetic map spanned 432.64 cM and consisted of 1318 windows of 20 DSNPs. For Volkamer lemon, the map consisted of 1016 markers spanning 425 cM. Both tetraploid Volkamer lemon and Swingle citrumelo genetics maps are more than two times smaller than the diploid trifoliate orange genetic map and most of the saturated genetic maps published for *Citrus* [59,74,76,77,78]. Our tetraploid maps are also half the size of the genetic map of the tetraploid clementine, which displayed a mostly tetrasomic inheritance with very low levels of preferential pairing [56]. 

Ahmed et al. (2020) stated that the high level of chromosome pairing in the allotetraploid giant key lime was the main factor resulting in the low apparent interspecific recombination between *C. medica* and *C. micrantha*, the two progenitors. Indeed, high preferential pairing strongly limits the proportion of gametes that can undergo interspecific chiasmata and, consequently, interspecific recombination. This conclusion is supported by our observation of chromosomes with marked differences for preferential pairing between Volkamer lemon and Swingle citrumelo. By comparing the genetic map and chromosomic preferential pairing between Volkamer lemon and Swingle citrumelo, a clear negative relation is found between genetic distance and preferential pairing. In chromosome 2, Volkamer lemon displayed a higher value of preferential pairing; thus, we observed a lower recombination rate and smaller genetic distance. In contrast, in chromosome 5, a null value of PP was identified in Volkamer lemon coupled with a higher recombination rate and larger genetic distance. For this chromosome, the apparent intergeneric recombination between *Citrus* and *Poncirus* was severely limited in citrumelo by preferential chromosome pairing.

Recombination rates in interspecific hybrids [79] may also be affected by the divergence between parental genomes, whatever the ploidy level. Decrease of recombination linked with genome sequence divergence has been described at the diploid level for several species [80,81,82,83,84] and suspected in *Citrus* from a comparative genetic mapping study between clementine and sweet orange [78]. The authors suggested that higher recombination rates in some genomic regions of sweet orange were associated with the interspecific heterozygosity of *C. reticulata/C. maxima*, which is higher in sweet orange than in clementine. 

We cannot discard that, in addition to the predominant preferential pairing, the divergence between *Citrus* and *Poncirus* or between *C. reticulata* and *C. medica* contributed to limiting the recombination rates for the tetraploid Swingle citrumelo and Volkamer lemon, respectively, even for gametes resulting from the homologous pairing. Comparative genetic mapping between diploid and tetraploid Swingle citrumelo and Volkamer lemon should allow this hypothesis to be tested. Some works revealed increased recombination in polyploid compared to diploid. Recombination between homologous chromosomes was more frequent in autotetraploid and allotetraploid *Arabidopsis* than in diploids [85]. Similarly, *Brassica* allotriploid hybrids (AAC) showed increased recombination between homologous A chromosomes compared to AA diploids [86]. In these two works, the increased recombination concerned homologous chromosomes. In contrast, in our work with interspecific doubled diploid, we can only see the interspecific recombination between heterologous chromosomes, the homologous chromosome being fully identical. Our conclusion of reduced recombination in allotetraploid concerns, therefore, only the interspecific recombination. In *Brassica*, while the diploid AA displays a classical marked decrease of recombination in pericentromeric areas, a homogeneous distribution of recombination all along the genome between the homologous A chromosome was observed in the AAC triploid [86]. In the case of our two interspecific doubled diploids, the recombination pattern displayed similar strong limitations in centromeric and pericentromeric regions, as already described in diploid citrus mapping studies [56,67,78].

### 3.4. Preferential Chromosome Pairing in Tetraploid Rootstock Resulting from Chromosome Doubling of Interspecific Hybrids Is Unfavorable for QTL Analysis

Genome-Wide Association Studies (GWAS) were performed by combining phenotyping and genotyping data at the gamete population level to identify and characterize genes involved in root growth and development. Preliminary results of GWAS analyses showed mostly weak signals. However, a dose effect of *Poncirus* and *C. medica* ancestors was identified at the end of chromosome 1 for the number of secondary roots and root surface. In Volkamer lemon gametes, a significant signal was detected for the root diameter trait at the beginning of chromosome 9. Furthermore, a weaker signal was also observed in the Swingle citrumelo gamete population for roots surface at the end of chromosome 5. *C. medica* haplotypes appeared unfavorable, while *P. trifoliata* haplotypes were favorable for all identified QTLs. Strong preferential chromosome pairing during meiosis of interspecific doubled diploid complicates QTLs discovery due to reduced recombination limiting genetic disparities between hybrids and resulting in a very important excess of heterozygous gametes compared to the two classes of homozygotes. More robust results could be obtained in such allotetraploid populations by increasing the number of individuals. Another option, even if it will not allow for analysis of the ploidy and allele doses effects, should be to analyze the architecture of targeted traits using diploid progenies resulting from hybridization between diploid Volkamer lemon and Swingle citrumelo. After validation at the tetraploid level, genetic markers linked to the identified QTL may be used for marker-assisted selection on the tetraploid progenies. 

### 3.5. Implication for Rootstock Breeding Programs

The ‘tetrazyg’ strategy has been used previously for rootstock breeding [49,50,51] and has been shown to be effective in generating interesting recombinant tetraploid progenies. Tetrazyg breeding can be performed using somatic hybrids or doubled diploids of preselected diploid rootstock arising from chromosome doubling in nucellar cells, such as the two parents used in this work. We took advantage of the very reduced rate of the apomixis of the tetraploid Volkamer lemon compared to other tetraploid rootstocks [3] to obtain numerous tetrazyg hybrids in the 4× Volkamer lemon × 4× Swingle citrumelo hybridization. Intergeneric hybridization between the genus *Poncirus* and *Citrus* is commonly performed for rootstock improvement because it allows for the combination of interesting characteristics of both genera. When using intergeneric hybrids such as Swingle citrumelo, citrange, and citrandarin for rootstock breeding, it is, therefore, essential to transmit the complementary favorable genes of the *Citrus* and *Poncirus* grandparents to the progenies. 

Chromosome preferential pairing has an effect on the transmission of parental heterozygosity. It is also strongly involved in reducing effective interspecific recombination and, thus, the genetic and phenotypic diversity of the hybrid population. In this study, we identified the high value of preferential pairing, leading mainly to an intermediate inheritance for both parents. This is an interesting feature in the case of rootstock breeding. Indeed, PP strongly limits the recombination rate, so a great part of the genetic value of the parents is transmitted to the tetraploid sexual progeny. The highly marked disomy observed in 4× Swingle citrumelo will allow retention of many genes of interest from the original Swingle citrumelo that required a long selection effort. 

In a recent study [58] of the *Poncirus* reference genome, various loci of interest were involved in CTV resistance [9], nematode resistance [8], and HLB tolerance [59]. Thirty NBS-encoding genes involved in these traits of interest were located in the *P. trifoliata* genome on chromosomes 6, 7, and 9. Thus, these chromosomes would play a very important role in transmitting resistance or tolerance to HLB, CTV, and nematodes [58]. The inheritances of these genes were studied within the tetraploid rootstock population. A total of 77.89% of the hybrids inherited at least one copy of the *P. trifoliata* genome for all regions of interest. These tetraploid hybrids may have inherited the trait of interest and will be submitted to further wide evaluation for resistance to abiotic and biotic stresses and agronomic behavior.

A vigorous root system is one of the main selection criteria in rootstock improvement programs. Previous studies suggested that, in the field, doubled-diploid rootstocks presented a superficial ramified root system and had less ability to capture soil resources during drought stress. We evaluated the root system architecture in our hybrids population in the present study. Significant phenotypic variability between hybrids was observed for all measured traits (number of secondary roots, root length, root surface area, and root diameter). Even though most of the hybrids presented a limited root development compared with diploid Citrumelo and Volkamer lemon, suggesting a limited capacity to go deep in the soil to absorb water, a limited number of hybrids presented root development higher or similar to Swingle citrumelo 2×, making these genotypes of interest for further evaluation.

## 4. Materials and Methods

### 4.1. Plant Material

Sexual hybridization between the tetraploid Volkamer lemon, SRA 1122 (*C. reticulata* × *C. medica*), as the female parent and tetraploid intergeneric Swingle citrumelo, SRA 1112 (*C. paradisi* × *P. trifoliata*), as a male parent was performed to obtain 103 tetraploid rootstocks hybrids. The two parents were selected several years ago among their diploid corresponding rootstock seedlings [37]. As described by [3], zygotic seedlings were selected by SNP marker genotyping, and tetraploidy of the 103 hybrids was confirmed by flow cytometry analysis of young leaves.

Other genotypes representative of the *Citrus* and *Poncirus* diversity were added to the study for diversity analysis, including three accessions representative of the trifoliate orange horticultural group (‘Flying Dragon’, ‘Pomeroy’, and ‘Kryder’ trifoliate oranges), one accession representative of the pummelo horticultural group (‘Seedless’ pummelo), two accessions representative of the citron horticultural group (‘Humpang’ and ‘Poncire commun’ citron), and two accessions representative of the mandarin horticultural group (‘Cleopatra’ and ‘Sunki’ mandarin) and the ‘Marsh’ grapefruit as a representative of *C. paradisi* (the second parent of the Swingle citrumelo). The Inrae-Cirad Citrus Biological Resources Center provided all these genotypes in San Giuliano, Corsica, France.

### 4.2. DNA Extraction and Genotyping by Sequencing (GBS)

Following the protocol of [69], genomic DNA was isolated using the Plant DNAeasy^®^ kit (Qiagen, Hilden, Germany) according to the manufacturer’s instructions. The concentration of genomic DNA was adjusted to 20 ng/μL, and ApeKI GBS libraries were prepared following the protocol described by Elshire et al. [87] and adapted to citrus by Ahmed et al. [67] with the use of a selective base (A) during the PCR step to reduce complexity as recommended by Sonah et al. [88]. Single-end and pair-end sequencing were performed on a double and single lane of an Illumina HiSeq4000.

### 4.3. SNP Calling

Raw sequencing data were demultiplexed using GBSX v1.1.2 [89] for paired-end reads and using Sabre (https://github.com/najoshi/sabre; accessed on 3 May 2022) for single-end reads. Cutadapt v1.8 [90] was used to remove the adapter sequences and low-quality bases (-q 20,20). Processed reads shorter than 30 were discarded. SNP calling and filtering were performed with the VcfHunter package described in [91] (https://github.com/SouthGreenPlatform/VcfHunter/; accessed on 3 May 2022) using VcfPreFilter.1.0.py and vcfFilter.1.0.py utilities. This procedure included mapping reads against the *P. trifoliata* reference genome (v1.3.1, https://phytozome.jgi.doe.gov/; accessed on 3 May 2022) using the BWA software [92]. For SNP calling, a minimal coverage by accession to keep genotype calling was set to 10, and a Minimal Allele Frequency (MAF) to keep genotype calling was set to 0.05. We only considered diallelic polymorphic positions. SNPs and genotype information (VCF file) were then imported into the SNiPlay3 application [93] for subsequent SNP exploration and analysis. The Illumina Hiseq sequencing raw data are available in the NCBI SRA (Sequence Read Archive) under the accession number PRJNA898312.

### 4.4. DSNPs Identification

Ancestral dose analysis along the genome was performed based on the number of allelic reads of ancestral diagnostic SNPs (DSNPs). According to the known phylogenomic structures of the parents, four ancestral species were considered: *P. trifoliata*, *C. medica*, *C. maxima*, and *C. reticulata*. Diagnostic SNPs for the ancestral species were selected using GBS data from eight accessions representative of the considered taxa: three for *P. trifoliata*, one for *C. maxima*, two for *C. medica*, and two for *C. reticulata*.

The search for diagnostic SNPs for the three *Citrus* ancestral species and *P. trifoliata* was based on the estimation of the inter-population differentiation parameter (*GST)* defined by [94]. For each taxon, *GST* estimation was performed considering two sub-populations: (1) the taxon concerned (*T_i_*) and (2) a theoretical population of the three other taxa (*T_−i_*). Analyses were performed from the estimated allele frequency of each taxon considering the same population size for each taxon to estimate the frequency of the two sub-populations (*T_i_* and *T_−i_*) and the frequency of the whole population (*Tot*):

(1) $$GST=\frac{He_{Tot}-\frac{\left(He_{T_{i}}+He_{T_{-i}}\right)}{2}}{He_{Tot}}$$

where *He* is the expected proportion of heterozygous loci per individual under Hardy–Weinberg equilibrium (*He* = 1 − Σ *p_i_*^2^, *p_i_* is the frequency of a given allele in the population or sub-population considered). *GST* values ranged from zero to one. *GST* = 1 means that the two taxa were fully differentiated and were the criterion for DSNP identification. 

Neighbor-joining and factorial analysis were computed using DARwin software version 6.0 [95]. Both were based on the Manhattan index:

(2) $$D_{i-j}=1/K\overset{K}{\underset{1}{\sum }}|x_{ik}-x_{jk}|$$

where *i* and *j* are the two considered individuals, *k* is the considered locus, *K* is the total number of loci, and *x_ik_* is the frequency of the alternative allele at locus *k* for the individual *i*. Three-dimensional (3D) visualization was done using the R package {rgl} [96].

### 4.5. Matrix Preparation

**Figure 7:**
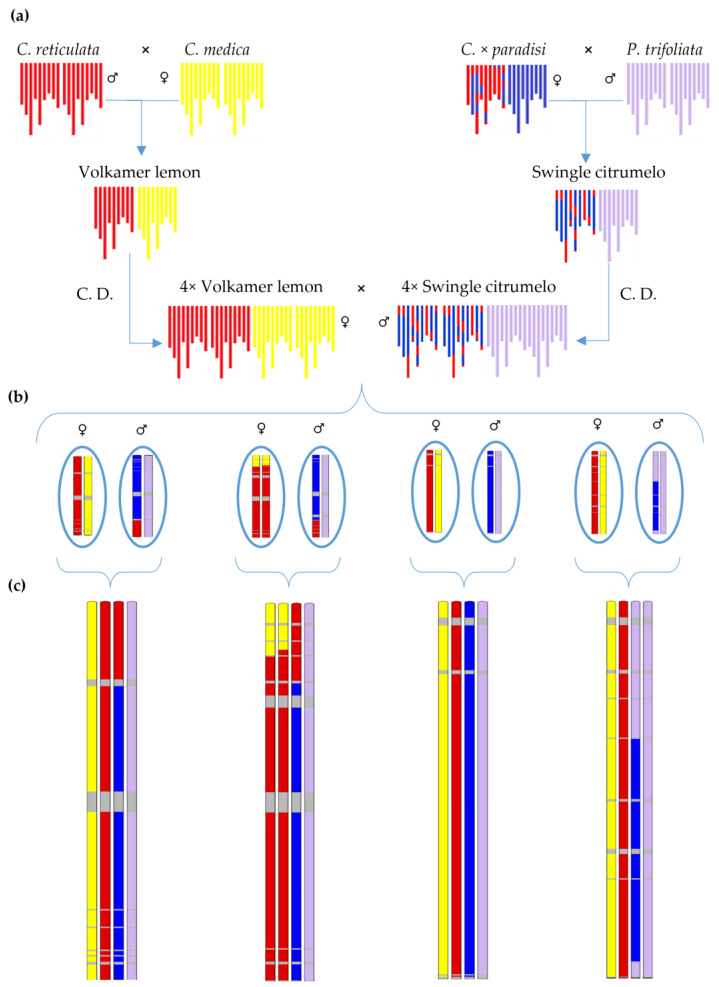
Phylogenomic structure of tetraploid parents and inference of phylogenomic structure of tetrazyg hybrids. (**a**) Origin and phylogenetic karyotypes of the tetraploid parents used in this paper. (**b**) Examples of Volkamer lemon ovule and Swingle citrumelo pollen phylogenetic structure for chromosome 1 and (**c**) examples of phylogenetic structure of chromosome 1 for four intergeneric Volkamer lemon × Swingle citrumelo tetraploid hybrids. C.D.: chromosome doubling of nucellar cells. Ancestral genome contribution: *C. reticulata* (red), *C. maxima* (blue), *C. medica* (yellow), *P. trifoliata* (purple), indeterminate area (grey).

A previous study [5] showed that polyploid allelic doses could not be estimated accurately at a single SNP locus from the GBS data generated with our protocol. Therefore, we developed an efficient approach based on the frequency of reads of phased successive markers to estimate the haplotypic doses in successive genomic windows of n markers. The published tool, called TraceAncestor, was successfully applied to analyze the phylogenomic structure of *Citrus* polyploid germplasm [5]. Our Volkamer lemon × Swingle citrumelo progeny has an adequate phylogenomic structure to infer allelic doses at the gamete level using the TraceAncestor tool and ancestral DSNP information. Indeed, Volkamer lemon is in *C. reticulata*/*C. medica* heterozygosity all along the genome, while the Swingle citrumelo genome alternates between *P. trifoliata*/*C. reticulata* and *P. trifoliata*/*C. maxima* heterozygosity. Therefore, estimating the allelic doses of *C. medica* and *P. trifoliata* in each tetraploid hybrid allows us to infer without ambiguity the phylogenomic structure of the corresponding Volkamer lemon ovule and Swingle citrumelo pollen (Figure 7).

The GBS data, the DSNP matrix, and TraceAncestor software were used to analyze the contribution of the four ancestral species along the genome of the intergeneric hybrids, following the method described by [5] using genomic windows of 20 DSNPs. We generated two matrixes from the hybrid mosaic structure: one for the Volkamer lemon parent with the dose estimation of *C. medica* for each window and another for the Swingle citrumelo parent with the dose estimation of *P. trifoliata*. Then, singletons were identified and replaced with missing data, as recommended by Van Os et al. [97], using a homemade Excel page routine. At the same time, a few individuals displaying an aberrant number of recombination, set by examining the global recombination distribution, were removed. These two matrixes were used for genetic mapping and the analysis of the meiotic behavior of each parent.

### 4.6. Mapping Analysis

The genetic map of the tetraploid Swingle citrumelo and Volkamer lemon was built using the {pergola} R package [98]. Genotyping data were filtered to less than 15% of missing data for markers and 20% for hybrids. 

The good quality assembly of *Poncirus*, the strong collinearity already demonstrated between the genus *Poncirus* and *Citrus* [3,59,73,74,75], and our choice to work on genomic windows of 20 successive markers led us to work based on physical ordering. In the present mapping analysis, we did not try to establish the order of the mapped windows, but we wanted to study the recombination rates along the genome.

The map was created using the multidimensional scaling algorithm and the Kosambi mapping function, allowing incomplete interference among the recombination events. The synteny and collinearity of both Swingle citrumelo and Volkamer lemon genetic maps with the reference *Poncirus* genome were visualized using Circos [99]; http://circos.ca (accessed on 25 February 2023), in Galaxy [100]. In addition, Marey maps were drawn using Microsoft Excel 2016 to visualize the collinearity between the genetic and physical position of the markers on the *Poncirus* reference genome [58]. 

### 4.7. Recombination Rate

Changes in the meiotic recombination rate along the genome are estimated with the online software « MareyMap Online » https://lbbe-shiny.univ-lyon1.fr/MareyMapOnline/ [101] accessed on 9 June 2022, with the following parameters: Loess estimation method with Span = 0.15. Recombination rates were estimated and visualized in Circos for the Swingle citrumelo and Volkamer lemon genetic map and the trifoliate orange genetic map generated by Huang et al. (2018; [59]).

### 4.8. Parental Heterozygosity Restitution (PHR)

The Parental Heterozygosity Restitution (PHR) was calculated at each locus as the percentage of individuals with the heterozygous allelic configuration. The estimation of PHR for each chromosome is the average of the values for all the markers on the chromosome.

### 4.9. Estimation of Preferential Pairing (PP)

Preferential Pairing (PP) defines the proportion of gametes resulting from the exclusive pairing of homologous chromosomes and ranges between 0 (full tetrasomic inheritance characteristic of autotetraploids) and 1 (full disomic inheritance in strict allotetraploids).

For a doubled diploid, under intermediate inheritance, marker segregation depends directly on the rates of chromosome Preferential Pairing (PP) and double reduction for the marker considered [56]. In the absence of recombination in the centromeric area and, hence, a null value for double reduction, there is a direct relationship between PP and PHR for the centromeric markers [56]. The PP values for three centromeric and peri-centromeric markers were estimated for each chromosome using the maximum likelihood approach proposed for centromeric loci by [56], and the average value was retained. Centromeric positions were estimated for areas with low recombination rates and gene density. For each locus, the estimation of PP was performed as follows:

Considering the tetrasomic parameter (τ = 1 − PP) that defines the proportion of gametes resulting from random meiotic chromosomal pairing used in several previous studies of meiotic behavior of tetraploid citrus [18,55,56,57,67,102] and according to [56], for centromeric loci, the probability of the three possible diploid gametes produced for an A1A1A2A2 duplex marker can be calculated using the following formulae:

P(A_1_A_1_) = τ/6 (3)

P(A_2_A_2_) = τ/6 (4)

P(A_1_A_2_) = 1 − τ/3 (5)

The probability of obtaining the observed gamete population as a function of τ is:

L(τ) = C × (P(A_1_A_1_)^x1^) × (P(A_2_A_2_)^x2^) × (P(A_1_A_2_)^x3^) = C × (1/6τ)^x1+x2^ × (1 − 1/3τ)^x3^ (6)

where C is a combinatory coefficient constant for the observed data and x1, x2, and x3 are the number of observed A_1_A_1_, A_2_A_2_, and A_1_A_2_ gametes. To estimate τ by a maximum likelihood approach, for each marker, we analyzed the L(τ) functions with τ values varying from 0 to 1 with a 0.005 interval. The estimated τ value was the one maximizing L(τ). PP was then calculated as 1 − τ.

R Studio V3.5.3 environment (R Studio Team, 2016, R Core Team, 2018, Vienna, Austria) was used for statistical analysis for PP and PHR. QQplot and a Shapiro–Wilk test were performed to test the normal distributions of PHR and PP. Normal distribution was confirmed for PP but rejected for PHR (*p*-value < 0.05). Analyses of variance (ANOVA) were performed to determine significant PP differences between chromosomes using a Newman–Keuls test for mean comparisons. A non-parametric Kruskal–Wallis test was used, followed by a Wilcoxon test, in order to study significant PHR differences between chromosomes.

### 4.10. Analysis of the Deviation from Expected Gametic Segregation under a Tetrasomic Model 

The same matrix used for genetic mapping was used to study the deviation from expected gametic segregation under a tetrasomic model without double reduction all along the genome for each parent. The *p*-values for the Chi2 test according to the tetrasomic theoretical frequency for each possible gamete (for a duplex locus A_1_A_1_A_2_A_2_: A_1_A_1_ = 1/6; A_2_A_2_ = 1/6; and A_1_A_2_ = 4/6) were computed with Excel, and we used the approach proposed by [103] to limit the False Discovery Rate (FDR) in multiple testing; the procedure was performed according to the method of [61] with a q-value threshold of 0.05. The results were visualized in a Circos plot.

### 4.11. Inheritance of Candidate Genes in the Hybrid Population

Loci associated with *Citrus trizteza virus* (CTV) resistance [104], nematode resistance (Tyr1) [8], and Huanglongbing (HLB) tolerance [59] were previously localized in *P. trifoliata*. In their study, [58] identified candidate genes in the *Poncirus* reference genome associated with these traits. The inheritance of these candidate genes was investigated in the tetraploid hybrid rootstock population. For this purpose, the positions of the genes were located on the genome, and the contribution of the *Poncirus* ancestor to this position was defined (zero, one, or two doses of *Poncirus* were administered to each hybrid). 

### 4.12. Root System Architecture Phenotyping

A phenotypic analysis of the root architecture of the hybrids was performed on four-month-old cuttings. The analyses were performed on 100 genotypes with 6 replicates per genotype. Photographs of the root system of each individual were taken and analyzed using ImageJ software [105] to determine the total root area, length of the longest root, number of secondary roots, and root diameter. Statistical analyses were then performed in R using the R Studio V3.5.3 environment (R Studio Team, 2016, R Core Team, 2018, Vienna, Austria).

### 4.13. Quantitative Trait Locus Analysis

We set up a strategy to evaluate the association between the allelic dose of the ancestors and the phenotypic information collected for the root system architecture. We took advantage of the fact that Volkamer lemon and citrumelo were fully heterozygous all along the genome for *C. medica* and *P. trifoliata*, respectively, to perform an association study between the doses (0, 1, or 2) of these two ancestors in the two gamete populations and the phenotypic traits of the tetraploid hybrids (root surface, root length, root diameter, and the number of secondary roots). We used the ancestor doses previously estimated with TraceAncestor in the successive windows of 20 ancestor diagnostic SNPs. We considered these window markers (positioned at the average value of the positions of the considered 20 DSNPs) to perform the association study using the GLM (General Linear Model) procedure under the default settings in TASSEL 5.0 [106].

## 5. Conclusions

In this study, we analyzed GBS data of a tetraploid hybrid rootstock population of 103 progenies from the cross between Swingle citrumelo and Volkamer lemon. Estimation of allelic doses of *C. medica* and *P. trifoliata* ancestors in each tetraploid hybrid allowed the deduction of the phylogenomic structure of gametes of both Volkamer lemon and Swingle citrumelo, respectively. Two high-density recombination landscapes and a meiotic behavior analysis of tetraploid Swingle citrumelo and Volkamer lemon were established. Concerning the *Poncirus* diploid genetic map previously published, the recombination rate was lower compared to tetraploid Swingle citrumelo and Volkamer lemon. This can be mainly explained by the high preferential chromosome pairing detected. The measured meiotic parameter is defined primarily as an intermediate inheritance with a high disomic tendency for Swingle citrumelo. In Volkamer lemon, a more complicated meiotic inheritance was highlighted with very different patterns between chromosomes, including tetrasomic segregation, intermediate inheritance with tetrasomic tendency, intermediate inheritance, and intermediate inheritance with disomic tendency. Significant variability among progenies was found for root architecture traits, but the substantial reduction of interspecific recombination by the preferential chromosome pairing affected the ability to detect QTL for root architecture. Conversely, the segregation pattern with high preferential pairing appears beneficial for rootstock breeding at the tetraploid level. Indeed, there is a high level of parental interspecific heterozygosity restitution, and the lower apparent recombination rate limits allele shuffling. Thus, a large part of the genetic value of the parents is transmitted to the descendants. In that way, we found that genome regions present in the *Poncirus* genome involved in CTV and nematode resistance and Huanglongbing tolerance had a strong level of inheritance in the offspring. Therefore, we can conclude that the “tetrazyg” strategy with interspecific doubled-diploid parents enhances the transfer of the genetic value selected at the level of initial diploid rootstock. The tetrazyg method also allows the generation of some tetraploid hybrids with vigorous root systems contrary to doubled-diploid rootstocks. It is, therefore, highly promising for rootstock breeding. Our tetrazyg progenies were recently propagated by cutting with the perspective to perform field evaluation of agronomic performances and evaluation for HLB tolerance. 

## Figures and Tables

**Table 1:** Distribution of the 72,452 diagnostic SNPs (DSNPs) per taxon and per chromosome.

Chromosome	*C. maxima*	*C. medica*	*C. reticulata*	*P. trifoliata*	Total
C1	1101	2220	1338	3028	7687
C2	1153	2654	1923	3724	9454
C3	1579	3938	2420	5024	12,961
C4	977	2221	1577	2962	7737
C5	1118	2193	1471	2970	7752
C6	885	1924	1325	2485	6619
C7	880	1975	1320	2626	6801
C8	935	2018	1319	2570	6842
C9	816	2087	1248	2448	6599
Total	9444	21,230	13,941	27,837	72,452
%	13.03	29.3	19.24	38.42	100

C1–C9: the nine chromosomes of the reference *P. trifoliata* genome (Peng et al. 2020; [58]); %: percentage of DSNPs for the taxon.

**Table 2:** Summary of tetraploid Swingle citrumelo and tetraploid Volkamer lemon genetic maps.

	Swingle Citrumelo		Volkamer Lemon	
LG	Mks/LG	LG Size cM	Mks/LG	LG Size cM
1	149	36.47	106	49.16
2	172	68.94	126	27.10
3	236	98.46	187	105.41
4	143	37.09	108	26.40
5	139	43.34	105	79.72
6	116	46.59	94	30.25
7	128	34.59	96	30.09
8	119	28.20	97	20.10
9	116	38.97	97	56.78
Total	1318	432.64	1016	425

LG: linkage group; Mks: number of markers; cM: centi morgan.

**Table 3:** Parameters of the meiotic behaviors of the tetraploid Swingle citrumelo and tetraploid Volkamer lemon.

Chromosome	Swingle Citrumelo		Volkamer Lemon	
	PHR (Rank)	PP (Rank)	PHR (Rank)	PP (Rank)
Chr1	0.889 ± 0.004 (a)	0.742 ± 0.007 (d)	0.826 ± 0.008 (a)	0.583 ± 0.019 (d)
Chr2	0.802 ± 0.006 (b)	0.625 ± 0.033 (b)	0.911 ± 0.008 (b)	0.81 ± 0 (f)
Chr3	0.790 ± 0.007 (c)	0.69 ± 0.054 (c)	0.719 ± 0.008 (c)	0.317 ± 0.061 (c)
Chr4	0.919 ± 0.004 (d)	0.85 ± 0.043 (e)	0.872 ± 0.005 (d)	0.548 ± 0.061(d)
Chr5	0.849 ± 0.011 (e)	0.848 ± 0.040 (e)	0.692 ± 0.008 (e)	0 ± 0 (a)
Chr6	0.890 ± 0.012 (adf)	0.925 (f)	0.869 ± 0.006 (d)	0.675 ± 0.012 (e)
Chr7	0.817 ± 0.003 (g)	0.502 ± 0.029 (a)	0.874 ± 0.007 (d)	0.663 ± 0.036 (e)
Chr8	0.933 ± 0.006 (h)	0.923 ± 0.050 (f)	0.897 ± 0.006 (f)	0.672 ± 0.057 (e)
Chr9	0.917± 0.003 (f)	0.76 ± 0.045 (d)	0.764 ± 0.008 (g)	0.25 ± 0.054 (b)
Whole genome	0.859 ± 0.004	0.763 ± 0.054	0.817 ± 0.005	0.502 ± 0.099

PHR: parental heterozygosity restitution; PP: preferential pairing. PHR rank based on Kruskal–Wallis and Wilcoxon test and PP rank based on ANOVA followed by a Newman–Keuls test. Values with different letters in brackets present significant differences.

**Table 4:** Deviation to the tetrasomic gametic segregation model in (**a**) Swingle citrumelo and (**b**) Volkamer lemon gametes.

(a) Chromosome	N	−log(q-Value)	N < −log(0.05)	%
Chr1	149	4.276 ± 0.149 (c)	149	100%
Chr2	172	1.874 ± 0.129 (ef)	124	72.09%
Chr3	236	1.634 ± 0.171 (f)	108	45.76%
Chr4	143	5.300 ± 0.115 (b)	143	100%
Chr5	139	3.345 ± 0.275 (d)	122	87.77%
Chr6	116	4.368 ± 0.378 (c)	108	93.10
Chr7	128	2.084 ± 0.102 (e)	120	93.75%
Chr8	119	5.628 0.201 (a)	119	100%
Chr9	116	5.097 ± 0.111 (b)	116	100%
Total	1318	3.492 ± 0.104	1109	84.14%
**(b) Chromosome**	**N**	**−log(q-value)**	**N > −log(0.05)**	**%**
Chr1	106	2.565 ± 0.178 (d)	94	88.68%
Chr2	126	4.700 ± 0.222 (a)	126	100%
Chr3	187	0.549 ± 0.087 (f)	18	9.63%
Chr4	108	3.738 ± 0.113 (c)	108	100%
Chr5	105	0.407 ± 0.098 (f)	7	6.67%
Chr6	94	3.594 ± 0.153 (c)	94	100%
Chr7	96	3.578 ± 0.210 (c)	96	100%
Chr8	97	4.346 ± 0.190 (b)	97	100%
Chr9	97	0.959 ± 0.157 (e)	24	24.74%
Total	1016	2.568 ± 0.113	664	65.35%

N: number of genomic windows; −log(q-value): segregation distortion to tetrasomic model estimation with 95% confidence intervals; N > −log(0.05): number of genomic windows with a segregation distortion to tetrasomic model above the significance threshold; %: percentage of significant value.

**Table 5:** Inheritance in the hybrids population of candidate genes (according to Peng et al., 2020, [58]) located in the *P. trifoliata* reference genome.

Gene ID	Trait	Location on *P. trifoliata* Reference Genome			Hybrids % with n Dose of *Poncirus*			
		Scaffold	Start	End	0	1	2	N
Ptrif.0006s1501.1	Huanglongbing tolerance	Scaffold 6	17843557	17847864	4.21	83.16	9.47	3.16
Ptrif.0009s1449.1		Scaffold 9	16743738	16750183	5.26	90.53	1.05	3.16
Ptrif.0009s1451.1		Scaffold 9	16761424	16771587				
Ptrif.0009s1453.1		Scaffold 9	16803290	16817272				
Ptrif.0009s1458.2		Scaffold 9	16966396	16974146				
Ptrif.0009s2550.1		Scaffold 9	16873308	16878288				
Ptrif.0009s2650.1		Scaffold 9	16878879	16881473				
Ptrif.0009s1595.1		Scaffold 9	19302619	19305258	4.21	88.42	2.11	5.26
Ptrif.0009s1596.1		Scaffold 9	19351433	19355794				
Ptrif.0009s1599.1		Scaffold 9	19383517	19386756				
Ptrif.0009s1600.1		Scaffold 9	19400932	19403616				
Ptrif.0007s1586.1	*Citrus tristeza* *virus* disease resistance	Scaffold 7	11780276	11787445	10.53	77.89	9.47	2.11
Ptrif.0007s1587.1		Scaffold 7	11823916	11826636				
Ptrif.0007s1590.1		Scaffold 7	11860949	11863630				
Ptrif.0007s1595.1		Scaffold 7	11908817	11912236				
Ptrif.0007s1378.1	Nematode resistance	Scaffold 7	9840644	9843519	11.58	78.95	7.37	2.11
Ptrif.0007s1394.1		Scaffold 7	9974384	9977152	11.58	76.84	9.47	2.11
Ptrif.0007s1395.1		Scaffold 7	9985962	9988577				
Ptrif.0007s1396.1		Scaffold 7	9990366	9991216				
Ptrif.0007s1398.1		Scaffold 7	10007442	10010437				
Ptrif.0007s1402.1		Scaffold 7	10043069	10045927				
Ptrif.0007s1404.1		Scaffold 7	10056890	10060283				
Ptrif.0007s1406.2		Scaffold 7	10065703	10067962				
Ptrif.0007s1411.2		Scaffold 7	10097497	10100154				
Ptrif.0007s1415.1		Scaffold 7	10123503	10126433				
Ptrif.0007s2703.1		Scaffold 7	10139209	10144561				
Ptrif.0007s1481.1		Scaffold 7	10687572	10691702	11.58	78.95	9.47	0
Ptrif.0007s1484.1		Scaffold 7	10729463	10732201				
Ptrif.0007s1501.1		Scaffold 7	10812404	10852651				
Ptrif.0007s1502.1		Scaffold 7	10860634	10863348				

Hybrids % with n dose of *P. trifoliata:* percentage of hybrids containing 0, 1, or 2 doses of *P. trifoliata* at the corresponding genes position. N: percentage of undetermined origin.

## Data Availability

The Illumina Hiseq sequencing raw data from the GBS library are available in the NCBI SRA (Sequence Read Archive) under the accession number PRJNA898312.

## Supplementary Materials

The following supporting information can be downloaded at: https://www.mdpi.com/article/10.3390/plants12081630/s1, Figure S1: Links between the position of markers on the (A) tetraploid Swingle citrumelo and (B) tetraploid Volkamer lemon genetic map (LGs) and on the chromosome assembly (Chr) of *P. trifoliata* genome (Peng et al. 2020, [58]); Figure S2: Marey map plot of the nine linkage groups of (A) Swingle citrumelo and (B) Volkamer lemon compared with the *P. trifoliata* reference genome (Peng et al. 2020, [58]); Figure S3: Graphical representations showing the meiotic behavior of the tetraploid Swingle citrumelo and tetraploid Volkamer lemon. PHR: parental heterozygosity restitution; PP: preferential pairing. Figure S4: Photographs showing root system diversity in three 4× Volkamer lemon × 4× Swingle citrumelo hybrids (a, b, c) and in 4× Volkamer lemon (d). Figure S5: PCA showing correlation between measured root traits in the 4× Volkamer lemon × 4× Swingle citrumelo population; Figure S6: Boxplots showing the *C. medica* and *P. trifoliata* ancestors dose effect on two root traits (number of secondary roots and root surface) for four significant markers of chromosome 1.
