# Supplementary material for: Meiotic Behaviors of Allotetraploid Citrus Drive the Interspecific Recombination Landscape, the Genetic Structures, and Traits Inheritance in Tetrazyg Progenies Aiming to Select New Rootstocks
Source: Plants (Basel). 2023 Apr 12;12(8):1630. doi: 10.3390/plants12081630 (PMC10146282; doi:10.3390/plants12081630)

Leny Calvez, Alexis Dereeper, Aude Perdereau, Pierre Mournet, Maëva Miranda, Saturnin Bruyère, Barbara Hufnagel, Yann Froelicher, Arnaud Lemainque, Raphaël Morillon and Patrick Ollitrault.

Meiotic behaviors of allotetraploid citrus drive the interspecific recombination landscape, the genetic structures, and traits in-heritance in tetrazyg progenies aiming to select new rootstocks.

Supplementary Material

Supplementary Figure S1. Links between the position of markers on the (**a**) tetraploid Swingle citrumelo (**b**) tetraploid Volkamer lemon genetic map (LGs) and on the chromosome assembly (Chr) of *P. trifoliata* genome (Peng et al. 2020, [58]).

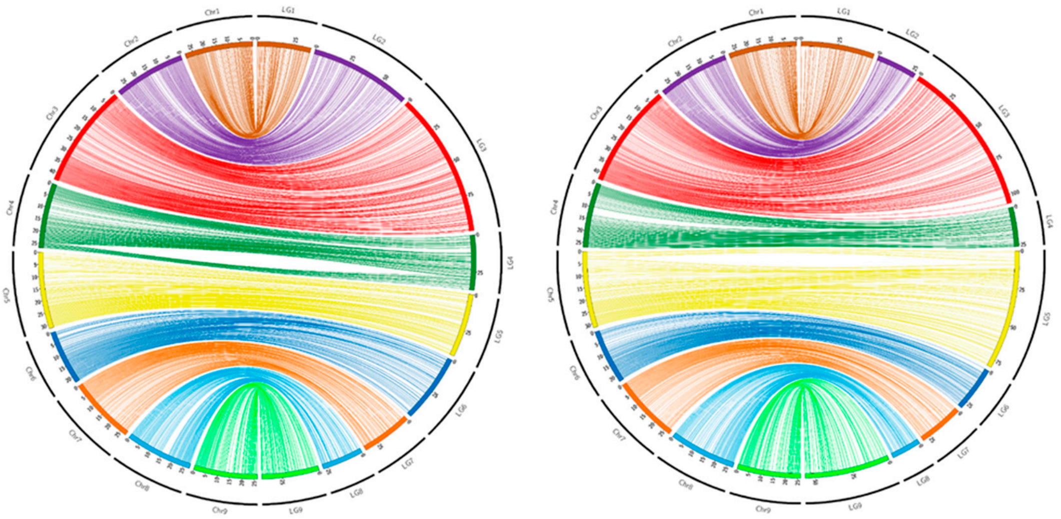

(a) (b)

Scales are in cM for LGs and in Mb for chromosomes.

Supplementary Figure S2. Marey map plot of the nine linkage groups of (**a**) Swingle citrumelo and (**b**) Volkamer lemon compared with the *P. trifoliata* reference genome (Peng et al. 2020, [58]).

(a)

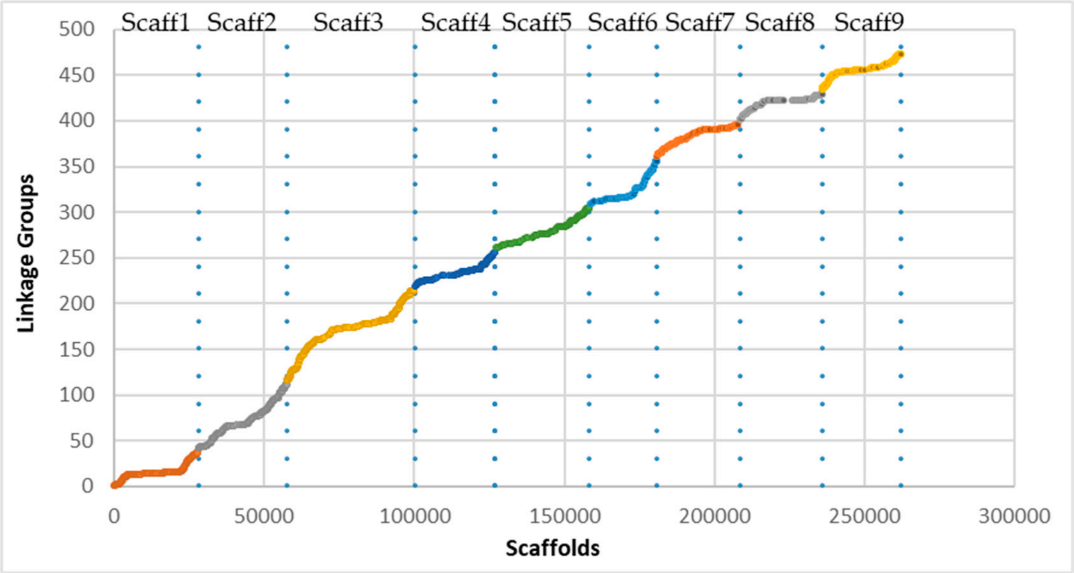

(b)

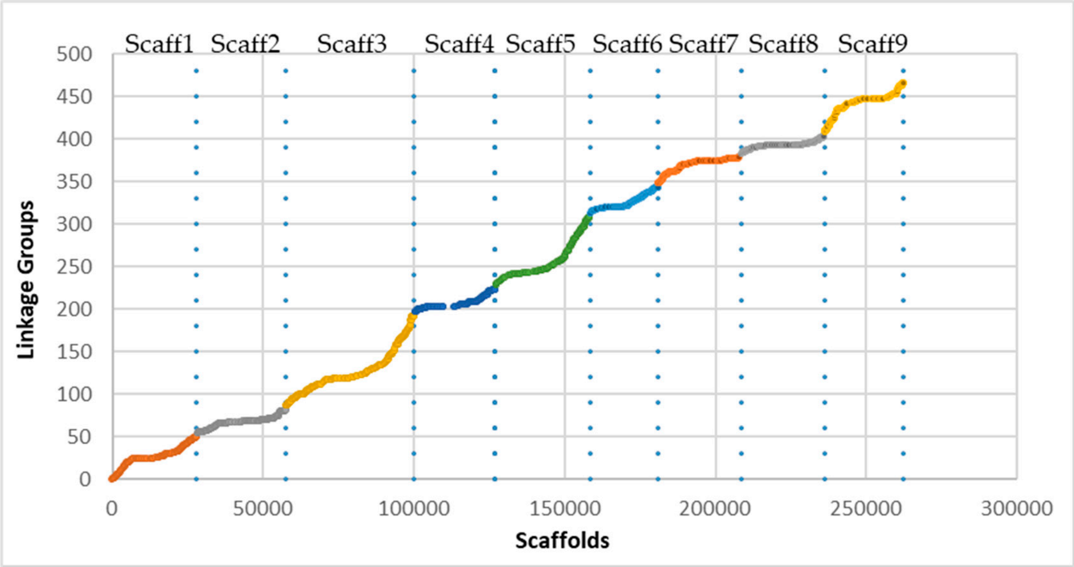

The x-axis represents the physical positions on the *P. trifoliata* reference genome and y-axis represents the position on the Swingle citrumelo and Volkamer lemon genetic maps.

Supplementary Figure S3: Graphical representations showing the meiotic behavior of the tetraploid Swingle citrumelo and tetraploid Volkamer lemon. PHR: Parental heterozygosity restitution; PP: Preferential pairing. (**a**) PHR of Swingle citrumelo (Kruskal-Wallis: *p*-value < 2.2e-16); (**b**) PHR of Volkamer lemon (Kruskal-Wallis: *p*-value < 2.2e-16); (**c**) PP of Swingle citrumelo (one-way ANOVA: *p*-value < 4.12e-15); (**d**) PP of Volkamer lemon (one-way ANOVA: *p*-value < 2.2e-16); (**e**) PP value from both Swingle citrumelo and Volkamer lemon population (two-way ANOVA: *p*-value < 2.2e-16).

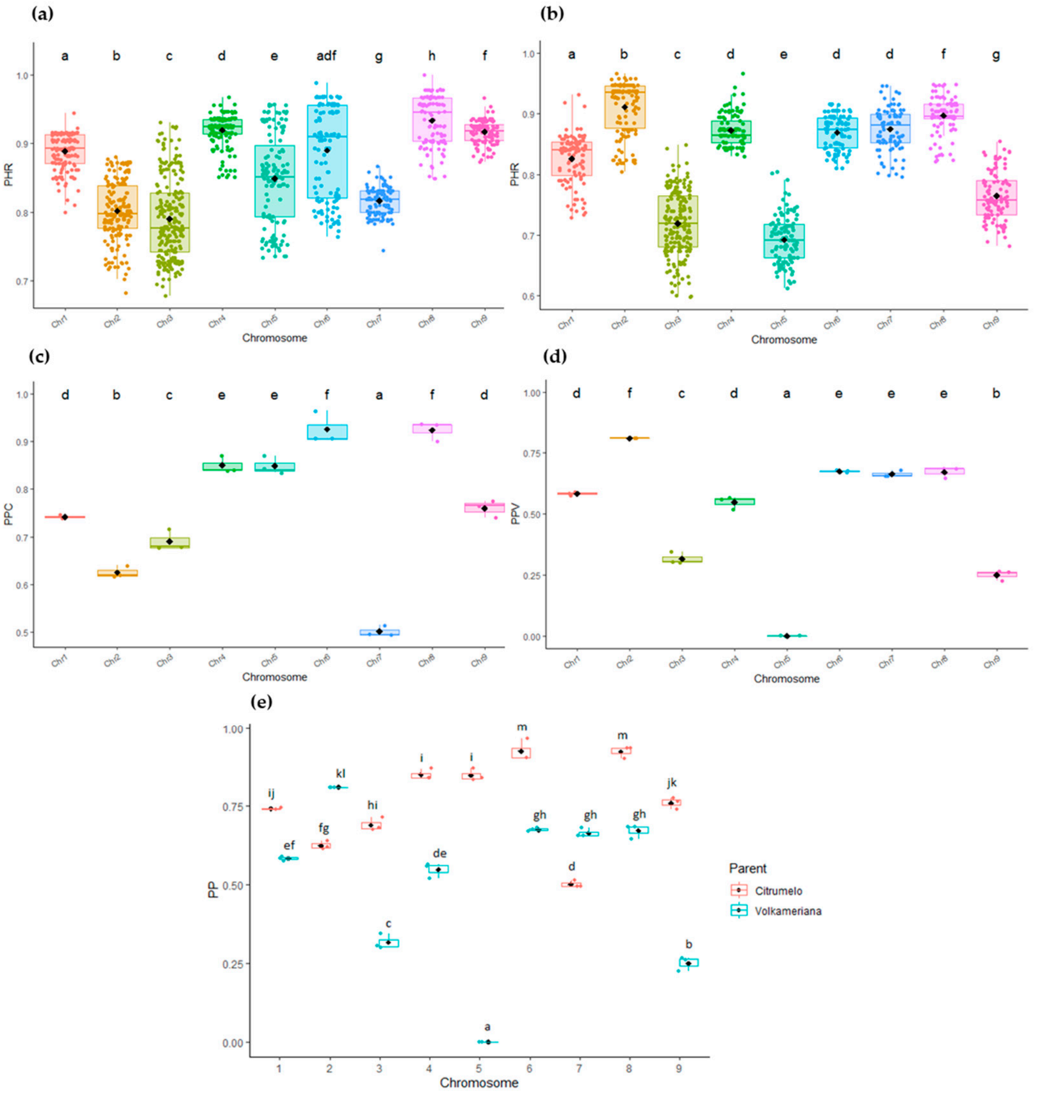

PHR ranks were estimated using a Wilcoxon and the PP rank using a Newman-Keuls test at a *p*-value < 0.05.

Supplementary Figure S4. Photographs showing root system diversity in three 4× Volkamer lemon × 4× Swingle citrumelo hybrids (**a**, **b**, **c**) and in 4× Volkamer lemon (**d**).

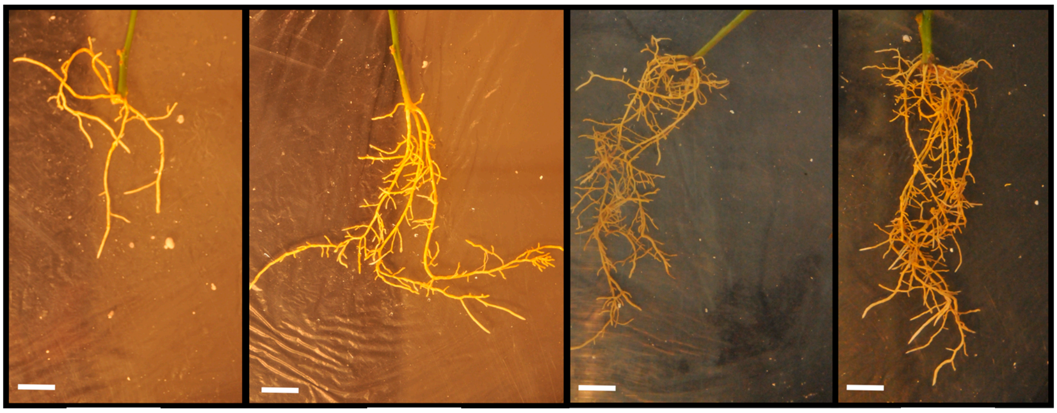

(a) (b) (c) (d)

Bars = 2 cm.

Supplementary Figure S5. PCA showing correlation between measured root traits in the 4x Volkamer lemon × 4× Swingle citrumelo population.

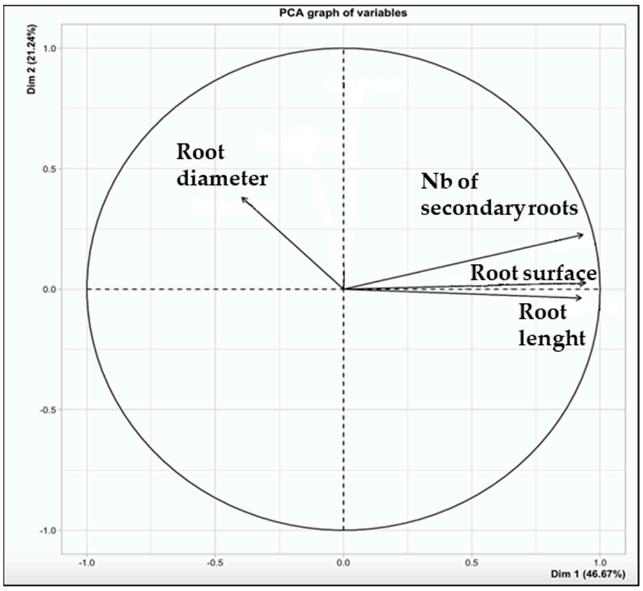

Supplementary Figure S6. Boxplots showing the *C. medica* and *P. trifoliata* ancestors dose effect on two root traits (number of secondary roots and root surface) for 4 significant markers of chromosome 1.

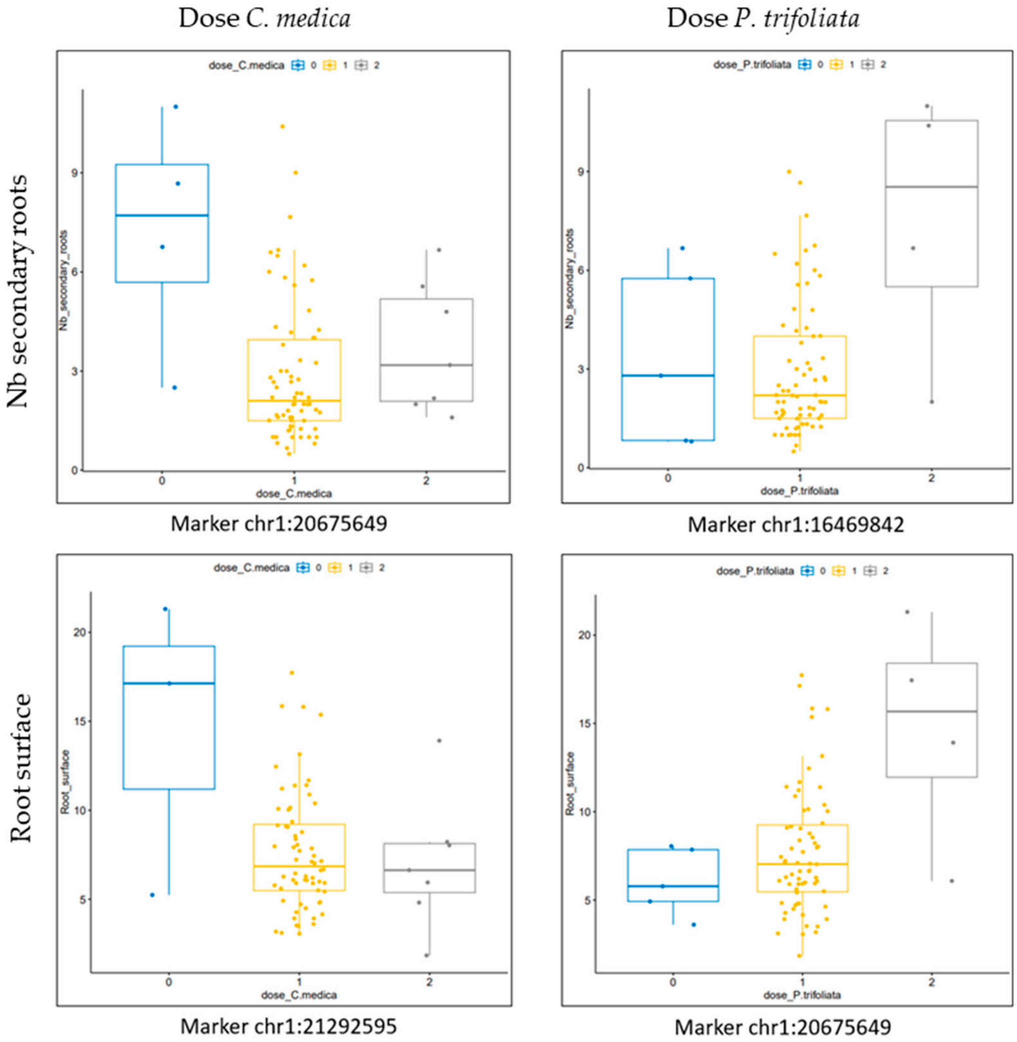

Supplement: Supplementary file 1 [file plants-12-01630-s001.zip › plants-2276271-supplementary.pdf]
